# *Ab Initio* Simulation of Structure and Properties in Ni-Based Superalloys: Haynes282 and Inconel740

**DOI:** 10.3390/ma16020887

**Published:** 2023-01-16

**Authors:** Wai-Yim Ching, Saro San, Caizhi Zhou, Ridwan Sakidja

**Affiliations:** 1 Department of Physics and Astronomy, University of Missouri Kansas City, Kansas City, MO 64110, USA; 2 Department of Mechanical Engineering, University of South Carolina, Columbia, SC 29208, USA; 3 Department of Physics, Astronomy and Materials Science, Missouri State University, Springfield, MO 65897, USA

**Keywords:** Ni-based superalloys, high entropy alloys, electronic structure, interatomic bonding, mechanical properties, total bond order density

## Abstract

The electronic structure, interatomic bonding, and mechanical properties of two supercell models of Ni-based superalloys are calculated using ab initio density functional theory methods. The alloys, Haynes282 and Inconel740, are face-centered cubic lattices with 864 atoms and eleven elements. These multi-component alloys have very complex electronic structure, bonding and partial-charge distributions depending on the composition and strength of the local bonding environment. We employ the novel concept of total bond order density (TBOD) and its partial components (PBOD) to ascertain the internal cohesion that controls the intricate balance between the propensity of metallic bonding between Ni, Cr and Co, and the strong bonds with C and Al. We find Inconel740 has slightly stronger mechanical properties than Haynes282. Both Inconel740 and Haynes282 show ductile natures based on Poisson’s ratio. Poisson’s ratio shows marginal correlation with the TBOD. Comparison with more conventional high entropy alloys with equal components are discussed.

## 1. Introduction

There is an increasing need for research on Ni-based superalloys [1] that can operate at higher temperatures because of their superior mechanical properties under supercritical steam environments [2,3,4]. Over the years, there has been remarkable progress in enhancing the creep properties to meet the stringent demands in materials properties designed for fossil energy (FE) power plants. In this regard research effort to discover new alloys that are capable of performing in aggressive environments is of prime importance. In particular, accurate theoretical modeling of their structure and properties at the atomic level can be instrumental in meeting these demands by providing fundamental understanding on their structure, composition, and interactions.

It is well known that Ni-based superalloys are heterogenous and contain many complex microstructures. However, it has been extremely challenging to construct microstructural-driven creep models without relying on a set of empirical parameters based on continuum mechanics. Indeed, such models have been very successful in representing the creep deformation for Ni-based superalloys [5,6,7]. There are two different approaches to tackle this problem, the “bottom-up” and the “top down” approaches. The “top-down” approach seeks the hidden correlations amongst the experimental setups which are usually overlooked. The data mining analyses can not only predict the creep properties but can also unearth the complex interdependency inherent within the creep database connecting the roles of chemical composition, heat treatment and mechanical properties [8,9]. The multidimensional interdependencies between various factors such as the variation in testing dimensions, chemical compositions and microstructures are very difficult to resolve quantitatively. The application of data mining toward the creep database has become ever more important for forging a stronger connection between the vast creep property database and multi-scale creep modeling with rigorous finite element analysis (FEA) [10,11]. A judicious selection process of the appropriate sets of creep models must be exercised [10,11,12]. The use of simple creep models in experimental protocols is not sufficient to accurately describe creep behaviors in high-strength superalloys with complex microstructures and voids.

We adopt the “bottom-up” approach via multimodal design, starting from the lowest level at atomic scale all the way to continuum level scale of real materials. This design potentially allows for the predictive capability of the model to be extended from the electronic to the continuum scale and to assess the mechanical property/deformation [13]. A common practice is that of periodic chemical modifications. Every new alloy explored will need new sets of empirical parameters to be onerously constructed. However, relying on experimental monitoring is too costly and unrealistic. Hence exclusive atomistic-scale modeling is the only choice despite the need for huge computational resources. This “bottom-up” design, in the context of a multi-modal approach, is capable of compartmentalizing the modeling and data gathering tasks. Thus the “bottom-up” approach has the capability to make predictions without exhaustive and time-consuming experimental probes.

There are many commercially developed Ni-based superalloys with registered trademarks from corporations such as HAYNES International and Special Metals Corporation. The two most common and advanced alloys are Haynes282 and Inconel740. The structure and composition of these commercial superalloys are very complex and are obtained by an onerous and expansive trial and error approach in production. This is motivated by the need to meet the demands of real applications, especially in the creep rupture, which can be collected from the vast amount of creep databases or from the open literature [14,15]. The names for these alloys originate from the way they were developed commercially.

The objective of this paper is to present our approach and the results of accurate atomic-scale modeling for Ni-based superalloys for use in power plants. There are virtually no such models for polycrystalline Ni-based superalloys, including Inconel740 and Haynes282. Thus, our overall goal is to establish a new portfolio in atomistic-based simulations that enables a deep fundamental understanding of these alloys. Results for Haynes282 and Inconel740 are presented side by side, not separately. No information has ever been reported on the electronic structure and interatomic interactions in these complex systems. We also discuss the relation of Ni-based superalloys with high entropy alloys (HEAs) [16,17,18], including their similarities and differences. This is the first time that such results are reported, providing valuable insights for future developments in Ni-based superalloys. Some of our earlier preliminary results on Ni-based superalloys are available for reference in [19].

## 2. Model Construction

### 2.1. Supercell Construction

**Figure 1:**
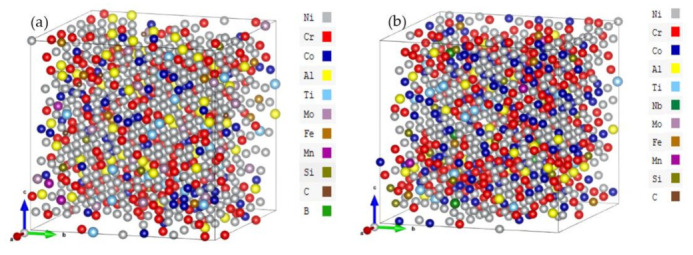
Sketch of supercell models of (**a**) Hay282 and (**b**) Inc740. The B atom is not shown since there is only 1 B atom in Hay282.

The cubic FCC supercells for Haynes282 (Hay282) () and Inconel740 (Inc740)) are modeled after converting their composition from the weight percentage to atomic percentage with 864 atoms each. The specific atoms are randomly distributed based on the random solid solution model (RSSM). The size of the supercell, or the number of atoms it contains is determined by the simple formula N = 4 × (*n*)^3^ for FCC cubic cell. In the present work, *n* is 6 so the supercell has 864 atomic sites. This size is sufficiently large to justify the use of the RSSM. The atomic numbers for each type of atom in the Hay282 and Inc740 models are listed in Table 1 and the supercell structures are depicted in Figure 1 after being fully optimized. The elements can be roughly divided into three groups. Firstly, the main transition metal (TM) elements (Ni, Cr, Co). Secondly, other TMs (Al, Ti, Nb, Mo, Fe, Mn)—Hay282 has no Nb and Inc740 has only 1 Mo. Finally, the nonmetallic elements (Si, C and B)—there is only 1 B in Hay282 and none in Inc740. Due to the presence of a variety of transition metal elements placed randomly, their distribution can be termed as highly inhomogeneous. In addition, both systems are dominated by Ni, hence their designations as Ni-based superalloys.

### 2.2. Computational Methods

Two different density functional theory (DFT) methods are used to perform the calculation for the Ni-based superalloys: Vienna ab initio simulation (VASP) [20,21,22] for structure optimization and mechanical properties and the orthogonalized linear combination of atomic orbitals (OLCAO) method [23] for electronic structure and interatomic bonding.

For VASP optimization, we use the standard projector augmented wave (PAW-PBE) potentials and generalized gradient approximation (GGA) for exchange–correlation potential. The following input parameters are used: kinetic energy cutoff of 500 eV, electronic convergence at 1.0 × 10^−5^ eV, and force convergence at −1.0 × 10^−3^ eV/Å and 1 k point at Γ in the reciprocal cell. For the elastic properties, we adopt the stress (*σ*_j_) vs. strain ($\epsilon_{j}$) scheme [24,25] on the VASP optimized structure for both Ni-based superalloys. A small strain of ϵ = ±0.5% is applied to the supercells to obtain the elastic coefficients *C_ij_* and their compliance tensor *S_ij_* (*i*, *j* = 1 to 6) by solving the set of linear equations:

(1) $$\sigma_{i}=\underset{j=1}{\sum }C_{ij}\epsilon_{j}$$

The OLCAO package [23] is an all-electron method with Gaussian type orbitals (GTO) that form atomic orbitals and use the local density approximation within DFT. The total bond order (TBO) of the optimized supercell is obtained by summing all bond order (BO) values for each atomic pairs $\;\rho_{\alpha \beta }$ defined in Equation (2). The BO value quantifies the bond strength between a pair of atoms with a specific bond length (BL). The total bond order density (TBOD) is obtained by dividing TBO by cell volume V. This quantum mechanical matrix is vital for describing the internal cohesion of the material since it contains both the BO values between all atoms and the optimized structure of the supercell. This novel concept has been applied to analyze many metallic systems [19] including HEAs [26,27,28].

Another important quantity from the OLCAO method is the effective charge $Q_{\alpha }^{*}$ Equation (3) on each atom. Both BO and the $Q_{\alpha }^{*}\;$ are based on the Mulliken analysis scheme [29] which requires a more localized minimal basis set. It should be pointed out that the Mulliken scheme is basis dependent.

(2) $$\rho_{\alpha \beta }=\underset{n,\;occ}{\sum }\underset{i,j}{\sum }C_{i\alpha }^{*n}C_{j\beta }^{n}S_{i\alpha ,\;j\beta }$$

(3) $$Q_{\alpha }^{*}=\sum_{i,j,\beta }\sum_{n,occ}C_{i\alpha }^{*n}C_{j\beta \;}^{n}S_{i\alpha ,j\beta }\;$$

Here *n, i, j,* $C_{j\beta }^{n}$, and $S_{i\alpha ,\;j\beta }$ are the band index, orbital quantum numbers, eigenvector coefficient, and overlap matrix, respectively. The charge transfer $\Delta Q^{*}$, also called partial charge (PC) of each atom is the deviation of $Q_{\alpha }^{*}\;$ from the neutral charge $Q_{\alpha }^{0}$.

The effective use of both VASP and OLCAO packages to investigate the structure and properties for large complex systems has been demonstrated in many materials including complex crystals, high entropy alloys (HEA) [26,27,28], biomolecular systems, and inorganic glasses [30]. However, this is the first time it has been applied to complex Ni-based superalloys.

## 3. Results

### 3.1. Atomic Structure

Table 2 lists the fully relaxed structure for Hay282 and Inc742 and their geometrical parameters, including cell volume and the average first nearest neighbor (1NN) and second NN (2NN). The differences between these two models are relatively minor and only reflect the difference in the composition of the metallic elements listed in Table 1. The fact that Hay282 has no Nb and Inc740 has no B has little influence in their atomic scale structures. Therefore, the differences between them must be explained in the context of the composition of the other elements that are present in both models, especially Ni, Cr and Co. These two Ni-based superalloys are fabricated commercially and on a purely trial-and-error basis with no theoretical guidance. In this respect, the compositional design for Ni-based superalloys is far more difficult to control than those in the high entropy alloys (HEA) which usually have equal or nearly equal metallic compositions. Hence high-level theoretical modeling is the only way to explain the origin of the specific properties of Ni-based superalloys and to contribute to their proper design.

**Figure 2:**
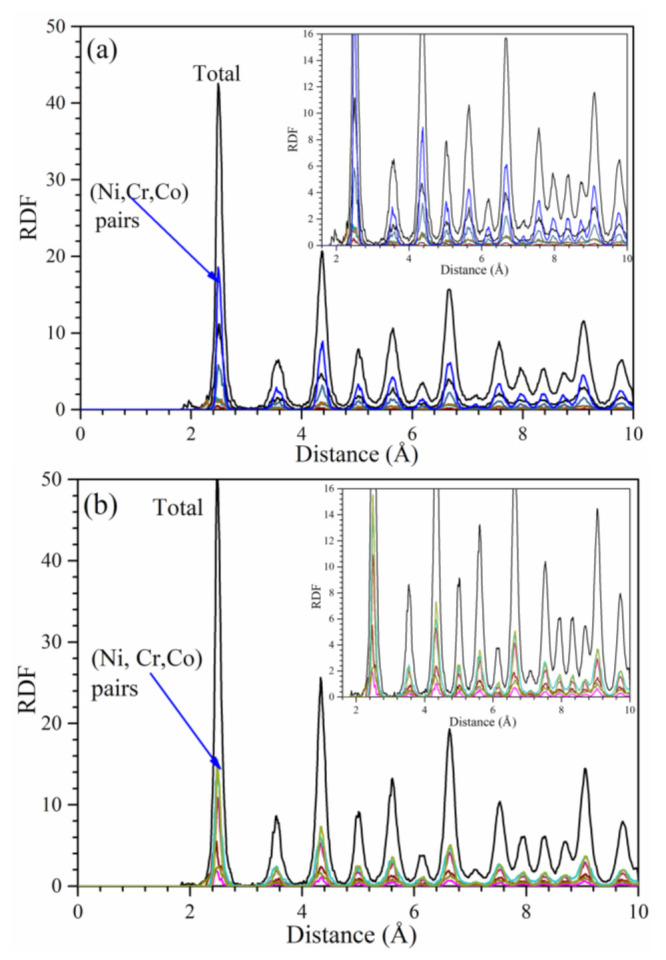
RDF and PRDF of (**a**) Hay282 and (**b**) Inc740. The PRDF are for (Ni, Cr, Co) pairs with negligible difference in peak positions with insets showing zoomed in sections of the graph for clarity.

The radial distribution function (RDF) and its partial components (PRDF) from 0 to 10 Å of Hay282 and Inc740 models are displayed in Figure 2a,b respectively. The differences in the PRDF for the (Ni, Cr, Co) pairs are negligible, which is consistent with the data in Table 2 for their first and second NN distance of separations.

### 3.2. Electronic Structure and Interatomic Bonding

**Figure 3:**
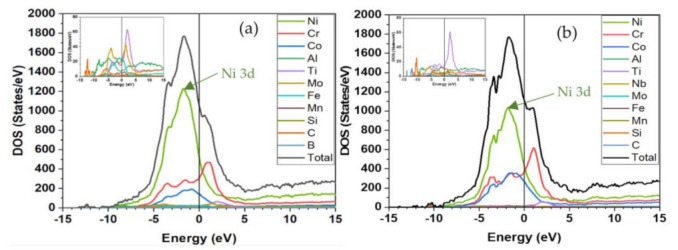
DOS and PDOS of (**a**) Hay282 and (**b**) Incon740. The inset shows the contributions from atoms with low participations.

The results for the electronic structure of Hay282 and Inc740 are expected to be similar to those in the atomic structures and to simply reflect the presence of different types of atoms. Hence, we will present only the results of the density of states (DOS) and partial DOS (PDOS), PC distributions, interatomic bonding, and, most importantly, the TBOD and PBOD. Figure 3 shows the comparison between TDOS and the atom-resolved PDOS of Hay282 and Inc740. The Fermi energy E_F_ is marked at the 0.0 eV. The E_F_ is located at the edge of a sharp peak, originating from the 3D orbital of the Ni atoms. Above E_F_, there is a small peak that can be traced to Cr atoms. The nonmetallic elements have negligible contributions and are mostly below the −10 eV. The values of the TDOS and its decomposition into PDOS at E_F_ (N(E_F_)) are listed in Table 3. The N(E_F_) has implications for the electric conductivity of metallic alloys, but is not discussed in the present work, because our focus is on the mechanical properties.

**Figure 4:**
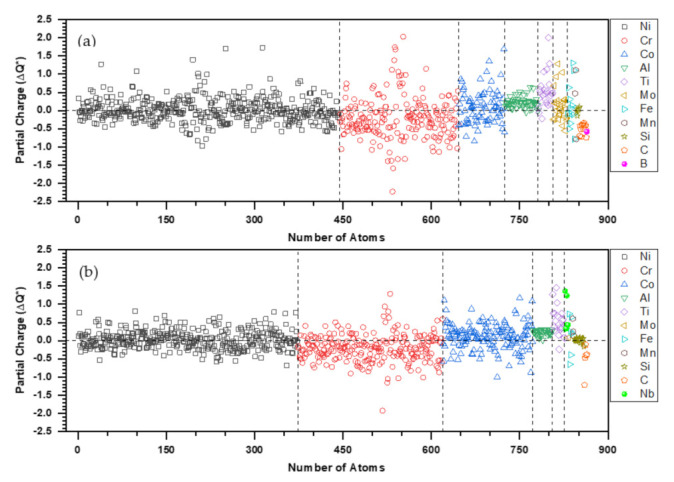
Atomic partial charge of (**a**) Hay282 and (**b**) Inc740.

Figure 4 shows the PC distribution for every atom in Hay282 and Inc740. The PC can be positive or negative for each metallic element. The range of deviation is larger in Hay282 than in Inc740. This reflects the difference in the atomic compositions and their interactions with their NNs in the two superalloys. As can be seen the larger and more scattered deviations are from Ni, Cr and Co.

Table 4 shows the summation of partial charge of different types of atoms in Figure 4.

**Figure 5:**
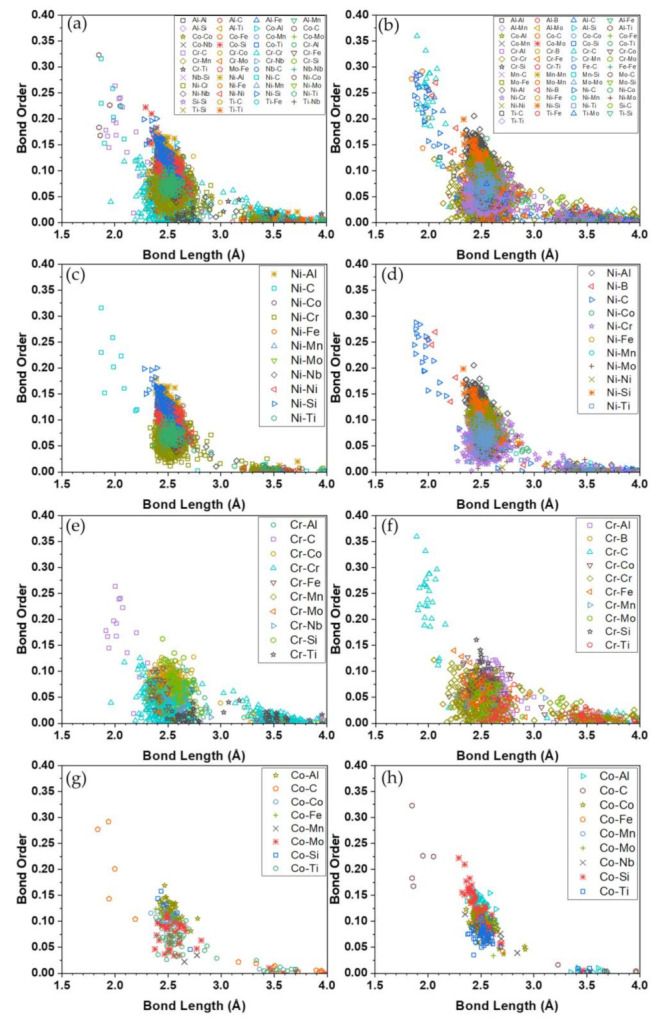
Bond order vs. bond length in Hay282 (Left panel: (**a**,**c**,**e**,**g**) and Inc740 (right panel: and (**b**,**d**,**f**,**h**).

Figure 5 shows the BO vs. BL distributions in Hay282 (left panel) and Inc740 (right panel). The top row ((a) and (b)) shows the data from all pairs, and the lower panels ((c) to (f)) show those involving the Ni, Cr, and Co atoms since they have the largest contributions to the TBOD as depicted in Figure 5. Most of the data points are clustered between 2.5 Å and 2.9 Å, the first NN of the metal atoms. The second NN pairs are the ones above 3.2 Å. The scattered pairs below 2.5 Å atoms mostly involve bonding with nonmetallic atoms, and they have larger BO values.

**Figure 6:**
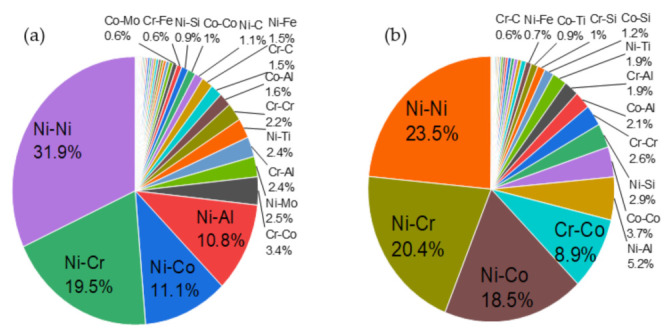
Pie chart for the pair contributions of: (**a**) Hay282 and (**b**) Incl740.

The Pie chart in Figure 6 displays the percentage of each pair to the TBOD in the two Ni-based superalloys from the data points in Figure 5. The top contributors are the Ni–Ni, Ni–Cr and Ni–Co pairs. This is followed by Ni–Al pairs (10.8%) in Hay282 and Cr-Co pairs (8.9%) in Inc740. Such detailed information on complex Ni-based superalloys has not been revealed before but is pertinent to the fundamental understanding of the superalloys physical properties.

### 3.3. Mechanical Properties

The calculated mechanical parameters of Hay282 and Inc740 from *C_ij_* and *S_ij_*, are listed in Table 5. They include the bulk modulus (K), shear modulus (G), Young’s modulus (E), and Poisson’s ratio (η) obtained using the Voight–Reuss–Hill (VRH) polycrystalline approximation [31,32,33]. In the VRH approximation, the elastic constantans are obtained from the average of the two calculations, one using maximum stress and the other using maximum strain. The mechanical parameters K, G, E and Poisson’s ratio η are obtained using the standard equations from *C_ij_*. The G/K ratio, also called Pugh ratio [34], is a useful parameter based on empirical arguments on polycrystalline samples [35]. It has been claimed that for G/K ratio greater (less) than 0.571, the material is more brittle (ductile). Table 5 shows that the G/K ratio for Hay282 and Inc740 are 0.350 and 0.369 respectively so they are both very ductile. It also shows that G/K marginally correlates with TBOD (Haynes 0.04274 and Inconel 0.04263). Vickers hardness Hv is estimated using the method proposed by Tian et al. [36].

**Figure 7:**
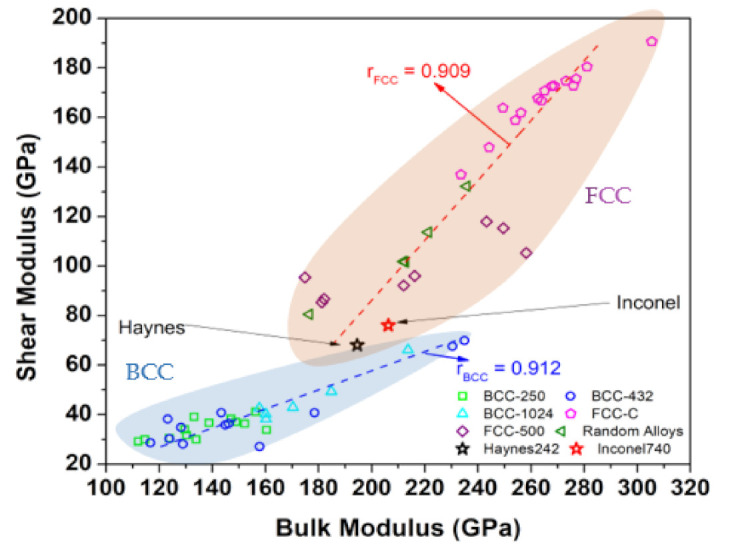
Comparison of calculated bulk modulus vs. shear modulus of Hay282 and Inc740 with different groups of HEAs.

In Figure 7, we compare the G/K ratio with the data for several HEAs, calculated using the same method as for Hay282 and Inc740. Details of the calculations for HEAs can be found in [26,27,28]. Figure 7 shows that the G/K for BCC and FCC HEAs are very different. For FCC HEAs, the G/K = 0.547 is obtained from the average of G/K for each data point and the correlation factor r = 0.909. For BCC HEAs the G/K = 0.256 is from the slope of the fitted line and the correlation factor is r = 0.912. The data for Hanes282 and Inconel740 (marked as open star) fall within the region of the FCC since the supercell models are constructed on an FCC lattice. The fact that they are approaching the region of the BCC HEAs can be attributed to the presence of the non-transitional metal elements in these two Ni-based supercells.

**Figure 8:**
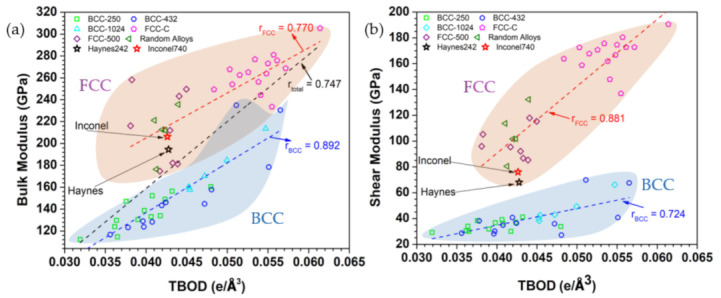
Correlation plot of Hay282 and Inc740 with different groups of HEAs: (**a**) bulk modulus vs. TBOD, (**b**) shear modulus vs. TBOD.

The correlations of the calculated TDOD with bulk modulus and shear modulus and different groups of HEAs are shown in Figure 8a,b, respectively, together with the data for Hay282 and Inc740. Both FCC and BCC HEAs have positive correlations between K vs. TBOD and G vs. TBOD. For bulk modules K, the correlation factor r is 0.770 and 0.892 for FCC and BCC, respectively. For shear modulus G, the correlation factor r is 0.881 and 0.724 for FCC and BCC, respectively. In order words, the degree of correlation is reversed in the case of shear modulus compared with the bulk modulus. The data points for Inconel and Haynes both stay at the lower region of the FCC HEAs, similar to that in Figure 7.

## 4. Discussion on the Nature of Metallic Bonding and Mechanical Strength

The Ni-based superalloys involve mostly metallic elements with some minor nonmetallic elements. This is even more complicated than those related to the theory of formation of HEAs or metallic glasses (MGs), because they do not contain traces of nonmetallic elements except as impurities or intentional doping. Most of the bonding types will certainly be attributed to metallic bonding. For example, the four metallic elements (Ni, Cr, Co, Al) that make up each alloy represent more than about 90% of the total composition. As much of the composition contributes to the formation of either a disordered FCC and/or an ordered FCC phase e.g., the L12 structure, we have employed the nominal composition as the basis for generating the alloy’s FCC representation. Metallic bonding is multiatomic in nature, different from covalent or ionic bonding where the bond length (BL) is explicitly defined as the distance between the two atoms forming the bond. In MGs, and to a lesser extent in HEAs, the precise assignment on the BL can be ambiguous, since all atoms within a certain distance of separation can contribute to metallic bonding. For a fixed distance of separation for a pair of atoms in the model, there could be many possible BO values, and for a specific value of BO, there could be many possible pairs of atoms with the same distance of separation. A theory that predominantly depends on the geometric parameter of BL, or on atomic size, for interpretation could be problematic. For Ni-based superalloys, these can be represented as “Ni-rich HEAs”, doped with a variety of nonmetallic elements. In any case, the concept of the TBOD is still applicable if the BO values of all the contributing atoms are counted. This point has already been strongly argued in reference [19] for MG. What differentiates HEAs and Ni-based superalloys from MGs is that HEAs have a basic crystal lattice with a face-centered cubic (FCC), body-centered-cubic (BCC), or hexagonal-close-packed (HCP) phase as its structural backbone. However, based on the RSSM description, HEAs or Ni-based superalloys are not necessarily made of simple crystalline phases. They possess more complicated long-range order (LRO) depending on the constituents presents in the alloys and may or may not contain short range order (SRO) in lieu of differing nearest neighbor (NN) and second nearest neighbor (SNN) pairs established by differing bond types generated locally. This is the same predicament that faces the vague description of the so-called lattice distortion, often ascribed to characterize the HEA’s internal crystalline structures [37]. In the current literature, the explanation for lattice distortion has focused mostly on the notion of an averaged valence electron count (VEC) of the constitutional metallic atoms. As shown in Figure 4 in the result section, the partial charge for the metallic atoms has a wide range of variations, not a specific number of electron configurations of metallic elements from the periodic table. Thus, it is more prudent to present the alloys within the context of a quantitative summation of the various bond types and bond strengths normalized to the equilibrium volume. The use of TBOD and its partial components, the PBOD, to explore the theory of formation in Ni-based superalloys is clearly a novel approach. As shown in Table 4, the TBOD values for the two Ni-based superalloys are quite comparable. This is to be expected, because both alloys are composed largely of metallic bonds from the three elements of Ni, Co, and Cr.

The addition of non-metallic alloying elements does contribute to the relatively minor differences on their overall mechanical strength. For example, in Table 4, the calculated bulk modulus of Inconel740 is slightly higher than that of Haynes282 (less than 7.5%). While our calculated bulk moduli for the two alloys are higher than reported by the industrial/manufacturer reports e.g., [38], the reports did indicate that the two alloys have a similar overall mechanical strength with Inconel740 to be slightly stronger than that of Haynes 282 (i.e., higher K and G), similar to our prediction. The overestimation of K and G may have stemmed from the redistribution of the non-metallic elements in the real alloys through precipitates and grain boundaries which we did not account for in our calculations. The Poisson’s ratio predicted by our calculations is comparable to those that have been previously reported (0.33–0.34 versus 0.31 for both alloys) and which are consistent with the largely metallic bonds from Ni, Co, and Cr -based pairs. These results explain why the two alloys are generally placed near to the bottom of the hypothetical HEA of FCC with the same types of elemental constituents as shown in Figure 7. Presumably, the larger K and G on the rest of the group plotted in Figure 7 were as a result of the contributions of semi-covalent bonds generated by TM–C pairs. As depicted in Figure 5, these pairs typically yield a much larger BO relative to those of metallic bonds.

Putting this altogether within the context of the overall strength of the crystalline structure shown in Figure 8, we note that the overall values of K and G of the two advanced superalloys are generally much lower than those that can be realized by the hypothetical FCC HEAs using the same exact constituents. Based on this plot, both the bulk and the shear modulus can be potentially improved almost 1.5 and 3 times respectively for the FCC HEAs. Certainly, we do not necessarily infer here that the mutual solubility limits of the other elements can be as high as those of Ni, Co, or Cr, and thus these hypothetical HEAs only serve as a guide. Nevertheless, this should provide the justification to develop superalloys further through the use of “HEA routes”. Additionally, we should also add that the typically lower strengths of the hypothetical BCC HEAs shown here can be simply understood from the fact that most of the metallic constituents of the two alloys can be stabilized as FCC structures, with the exceptions of Nb, Mo and Ti [39]. Overall, our results may lead to a potentially new direction as an alloying route map to develop the gamma (FCC) matrix phase of superalloys with superior mechanical strengths.

## 5. Conclusions

In this article, we present the results of the first principles calculation of the electronic structure, interatomic bonding, and mechanical properties of two Ni-based superalloys, Haynes282 and Inconel740. To the best of our knowledge, this is the first time such a detailed and fundamental analysis has been undertaken on the most popular commercial alloys used in the industry. Though Inconel740 and Haynes282 have similar compositions, their difference can be seen in the larger deviation in partial charge of Haynes282. In addition, mechanical properties and TBOD also show differences in these two Ni-based superalloys.

The special merits of using TBOD and PBOD as key metrics in assessing the fundamental properties of multicomponent alloys, including HEAs and metallic glasses, have been pointed out. This characteristic is different from other computational techniques based on ground state energies used in the enthalpy evaluation. The total energies for different HEAs can be very different unless the formation energy for each HEA is explicitly calculated, which is onerous for multi-component HEAs with different compositions. Moreover, the PBOD resolved from the TBOD have different pairwise components that are particularly useful in revealing the details of interatomic interactions, because TBOD and PBOD are derived from quantum mechanical calculations, not from geometric parameters. TBOD is further positively correlated with mechanical properties and Poisson’s ratio. Finally, the current approach can be extended to address other more challenging problems such as creep resistance, the role of grain boundaries, microstructures, and other types of defects discussed in the introduction.

## Figures and Tables

**Table 1:** Atomic percentage with the number of atoms for the two supercell models.

Element	Hay282		Inc740	
	Atom%	No.	Atom%	No.
Ni	51.37	444	43.13	373
Cr	23.42	202	28.46	246
Co	9.11	79	17.72	153
Al	6.53	56	3.81	33
Ti	2.90	25	2.41	21
Nb	--	--	0.71	6
Mo	2.92	25	0.17	1
Fe	1.35	12	0.69	6
Mn	0.31	3	0.31	3
Si	0.61	5	1.95	17
C	1.33	12	0.64	5
B	0.15	1	--	--
Total		864		864

**Table 2:** Relaxed structure of Hay282 and Inc740 with the 1NN and 2NN.

Model	a (Å)	b (Å)	c (Å)	α	β	γ	Vol(Å^3^)	1NN(Å)	2NN(Å)
Hay282	21.35	21.54	21.42	90.05	90.04	90.05	9850.61	2.53	3.57
Inc740	21.36	21.23	21.36	89.92	89.98	90.08	9686.95	2.51	3.55

**Table 3:** Comparison of DOS values at the Fermi level N(EF) for each type of atoms Hay282 and Inc740 in unit (states/eV).

Model	Ni	Cr	Co	Al	Ti	Nb	Mo	Fe	Mn	Si	C	B	Total
Hay282	524.78	307.41	143.82	8.02	17.91	--	22.4	22.68	4.86	0.87	2.02	0.18	1054.93
Inc740	432.12	368.22	268.62	4.39	14.53	4.11	0.76	10.3	4.27	2.58	0.97	--	1110.87

**Table 4:** Sum of effective charge (Q*) and partial charge (PC) in unit of electrons of different types of atoms in Figure 4.

Inconel740				Haynes282			
Element	Atoms	Q*	PC	Element	Atoms	Q*	PC
Ni	373	9.972	0.028	Ni	444	9.981	0.019
Cr	246	6.207	−0.207	Cr	202	6.213	−0.213
Co	153	8.885	0.115	Co	79	8.890	0.110
Al	33	2.779	0.221	Al	56	2.776	0.224
Ti	21	3.479	0.521	Ti	25	3.471	0.529
Nb	6	10.312	0.688	Mo	25	5.880	0.120
Mo	1	5.926	0.074	Fe	12	7.860	0.140
Fe	6	7.866	0.134	Mn	3	6.733	0.267
Mn	3	6.675	0.325	Si	5	3.995	0.005
Si	17	3.985	0.015	C	12	4.498	−0.498
C	5	4.514	−0.514	B	1	3.577	−0.577

**Table 5:** Comparison of the elastic properties and elastic moduli in the unit of (GPa) of Hay282 and Inc740, and TBOD in (eV/Å^3^).

Model	C_11_	C_12_	C_44_	K	G	E	ƞ	G/K	Hv	TBOD
Hay282	244.61	174.89	106.36	194.55	68.04	182.82	0.343	0.350	5.530	0.04277
Inc740	262.82	182.85	116.95	206.25	76.06	203.21	0.336	0.369	6.355	0.04263

## Data Availability

All data in this paper are presented in the form of figures and tables. No numbers generated in the calculations will be deposited since they involve more than one thousand lines and have to be recalculated on demand. Anyone who is interested in the specific numbers from the figures can contact the corresponding author (chingw@umkc.edu) directly.
